# Safeguarding the Ureter: Strategies to Prevent Intraoperative Ureteric Injury in Gynaecological Surgery

**DOI:** 10.7759/cureus.108094

**Published:** 2026-05-01

**Authors:** Madhumanti Sarkar, Pinky Jena, Vijyeta R Jagtap

**Affiliations:** 1 Obstetrics and Gynaecology, All India Institute of Medical Sciences, Rajkot, Rajkot, IND

**Keywords:** broad ligament fibroid, cervical fibroid, double-j stent, gynaecological surgery complications, iatrogenic ureteric injury, intraoperative ureteric identification, prophylactic ureteral stenting, retroperitoneal dissection, ureteric injury prevention, ureteroureterostomy

## Abstract

Iatrogenic ureteric injury is an uncommon but serious complication of gynaecological surgery, with significant potential for renal impairment, fistula formation, prolonged hospitalisation, and medico-legal consequences. The ureter is at risk not only in complex pelvic procedures but also during apparently straightforward operations, and injuries may go unrecognised at the time of surgery, contributing to delayed diagnosis and worse clinical outcomes.

We present a case series of five female patients aged 33, 59, 45, 46, and 47 years, respectively, who underwent complex gynaecological surgery for large fibroids at the Department of Obstetrics and Gynaecology, All India Institute of Medical Sciences (AIIMS), Rajkot, Gujarat, India. Each case illustrates a distinct clinical challenge and a tailored ureteric safeguarding strategy. Preoperative magnetic resonance imaging (MRI) fibroid mapping was employed. In one case with a large cervical fibroid and documented bilateral ureteric dilatation, contrast-enhanced computed tomography (CT) with three-dimensional ureteric reconstruction provided an informed surgical roadmap and guided the decision for bilateral prophylactic double-J (DJ) ureteric stenting. In another case, attempted left-sided preoperative stenting failed due to fibroid-related ureteric obstruction - a finding that was reinterpreted as a diagnostically valuable signal of significant ureteric compromise rather than a procedural failure, directly heightening intraoperative vigilance. Across all five cases, systematic retroperitoneal dissection with early identification and tracing of the ureter at the pelvic brim served as the consistent intraoperative safeguard. In a further case, despite preparedness and awareness, inadvertent left ureteric transection occurred during haemostasis from an extremely vascular broad ligament fibroid mass; the injury was recognised immediately, urological assistance was summoned intraoperatively, and successful ureteroureterostomy with DJ stent insertion was performed, with complete patient recovery.

Based on this experience, we propose a structured six-step protocol for ureteric safeguarding in complex gynaecological surgery: (1) preoperative risk stratification, (2) targeted imaging, (3) selective prophylactic ureteric stenting in cases with hydronephrosis or significant ureteric displacement, (4) systematic retroperitoneal dissection with early ureteric identification, (5) adjunctive intraoperative cystoscopy, and (6) preparedness for immediate surgical repair. This series demonstrates that a multimodal, anatomy-guided approach can substantially reduce the risk of ureteric injury in high-complexity cases, and that when injury occurs despite precautions, immediate intraoperative recognition and definitive repair are the most critical determinants of outcome.

## Introduction

Iatrogenic ureteric injury is an uncommon but serious complication of gynaecological surgery, associated with significant postoperative morbidity, loss of renal function, fistula formation, prolonged hospitalisation, and substantial medico-legal implications. The anatomical proximity of the ureters to the uterus, cervix, adnexa, and pelvic sidewall renders them particularly vulnerable during pelvic procedures, especially hysterectomy and complex benign or oncological surgeries [1].

Risk factors for ureteric injury have been consistently identified across large observational series and include previous pelvic or abdominal surgery, endometriosis or pelvic inflammatory disease, large uterine or cervical fibroids, pelvic malignancy, obesity, intraoperative haemorrhage or difficult dissection, and limited surgical experience. Importantly, several studies note that a significant proportion of ureteric injuries occur during procedures considered routine, underscoring the importance of vigilance even in apparently low-risk cases [2]. Large fibroids - particularly broad ligament and cervical fibroids - pose a particularly high-risk surgical challenge by displacing the ureter from its expected anatomical course and obscuring standard operative landmarks. Large cervical and broad ligament fibroids displace the ureter medially and cranially from its expected course, obliterating the surgical landmarks at the infundibulopelvic ligament and uterine artery crossing - the two anatomical zones of highest ureteric vulnerability [1].

The reported incidence of ureteric injury during gynaecological surgery varies widely by procedure type and complexity. Vaginal hysterectomy carries the lowest risk at approximately 0.02%-0.06%, while radical gynaecological oncology surgery carries the highest reported rates, reaching 10.7% in large national cohort data [3-9]. Institutional audits from India report ureteric injury rates of approximately 0.7%, reinforcing that such injuries remain relevant in high-volume tertiary care settings [9]. Laparoscopic hysterectomy has consistently been associated with a higher risk of ureteric injury compared to vaginal or abdominal approaches, particularly during the learning curve and in cases with distorted pelvic anatomy [7,10].

Although relatively infrequent, ureteric injuries are clinically significant because up to 60%-70% remains unrecognised intraoperatively, resulting in delayed diagnosis and worse outcomes (infection, urinoma, ureteric stricture, urinary fistula, and renal loss) [11].

This case series (n=5) from a tertiary care centre in India describes ureteric safeguarding strategies in complex gynaecological surgery, including preoperative magnetic resonance imaging (MRI) fibroid mapping, CT-based three-dimensional ureteric reconstruction (applied selectively), prophylactic double-J (DJ) ureteric stenting (in indicated cases), and systematic intraoperative retroperitoneal dissection. We also present one case in which, despite preparedness, intraoperative ureteric transection occurred, was immediately recognised, and was repaired definitively, illustrating the complementary importance of prompt intraoperative recognition alongside preventive strategies.

## Case presentation

Case 1

Giant Subserosal Fibroid: MRI Mapping and Retroperitoneal Dissection

**Figure 1:**
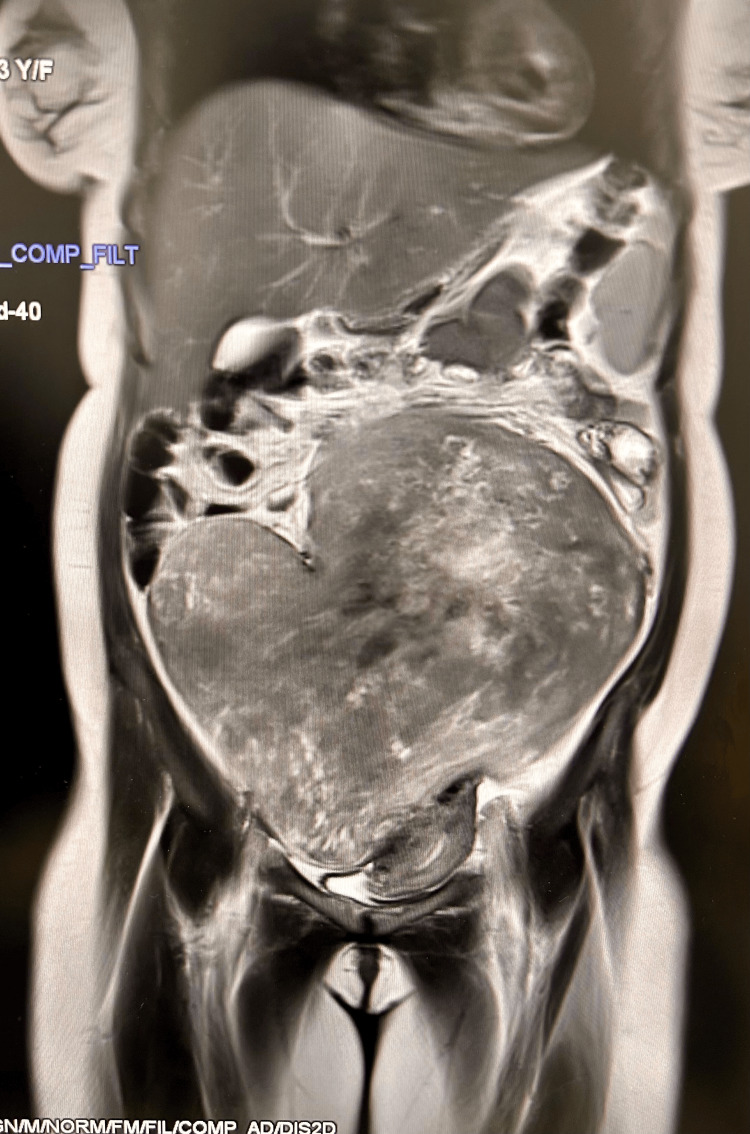
Case 1: MRI pelvis T2 sequence showing 13 × 20 × 22.5 cm mass A heterogeneously isointense mass arising from the right parametrium fills the entire pelvis, displacing the uterus to the left. MRI findings were suggestive of neoplastic aetiology, driving the decision for intraoperative frozen section.

A 33-year-old woman (P2L2A1) presented with lower abdominal heaviness, distension, and dyspepsia for six months. Ultrasonography showed a heterogeneous hyperechoic lesion (22.5 × 13.1 cm) in the right adnexal region, suggestive of a broad ligament or subserosal fibroid. MRI of the abdomen and pelvis confirmed a well-defined, heterogeneously T1/T2 isointense lesion (13 × 20 × 22.5 cm; Figure 1) arising from the right parametrium, with heterogeneous post-contrast enhancement, restricted diffusion (low ADC), and susceptibility artefact (blooming on gradient-recalled echoes (GRE)). The MRI findings raised concern for neoplastic aetiology, with broad ligament leiomyosarcoma considered more likely than leiomyoma, necessitating intraoperative frozen section. Additional findings included a right ovarian haemorrhagic cyst (2.7 × 1.7 cm) and left renal mid-calyceal calculi with focal hydronephrosis.

Given the size of the mass and its origin from the right broad ligament, with the MRI demonstrating significant displacement of the uterus to the left and proximity of the mass to the right pelvic sidewall, ureteric injury was identified as a significant intraoperative risk. Preoperative haemoglobin optimisation was performed with blood transfusion.

**Figure 2:**
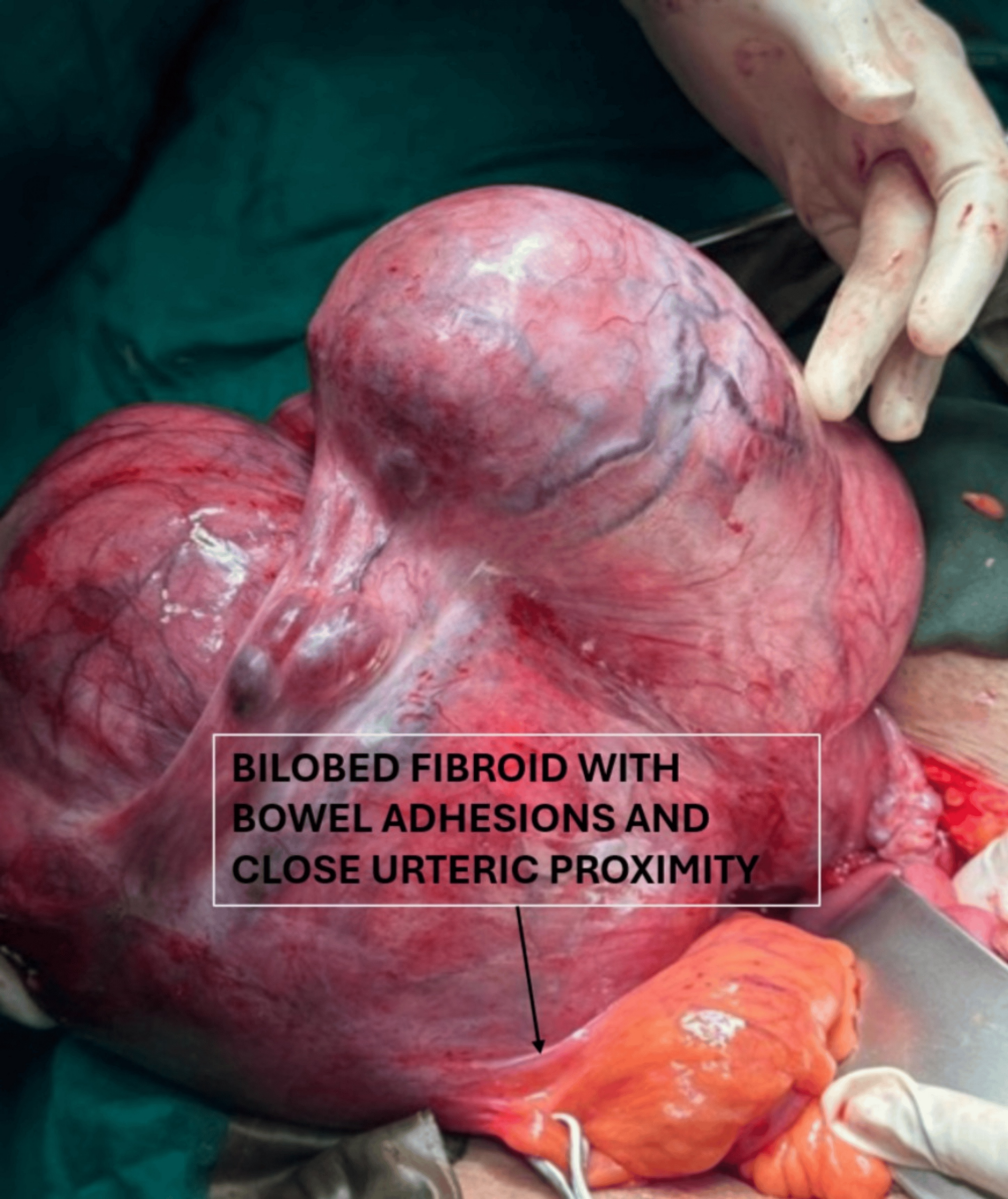
Case 1: Intraoperative photograph posterior view This figure shows the mass in situ with the surrounding viscera visible. The proximity of the mass to the right pelvic sidewall and bowel underscores the complexity of this dissection. Retroperitoneal identification of the right ureter was the key protective manoeuvre.

At open myomectomy, intraoperative findings revealed a 30 × 24 × 20 cm bilobed, soft, highly vascular mass arising from the posterior aspect of the fundus of the uterus. The key ureteric safeguarding strategy employed was systematic retroperitoneal dissection: the retroperitoneum was entered through the space lateral to the right infundibulopelvic ligament, and the right ureter was identified at the pelvic brim, traced distally, and kept under continuous direct visualisation throughout the dissection. No prophylactic DJ stent was inserted in this case, as retroperitoneal anatomical identification was deemed sufficient given the ureteric course on imaging. Frozen section confirmed leiomyoma with hydropic changes, and the decision was taken for myomectomy only, with preservation of the uterus and right adnexa. No ureteric injury occurred. The intraoperative photograph demonstrating the bilobed mass in situ with its proximity to the right pelvic sidewall and bowel is shown in Figure 2.

**Figure 3:**
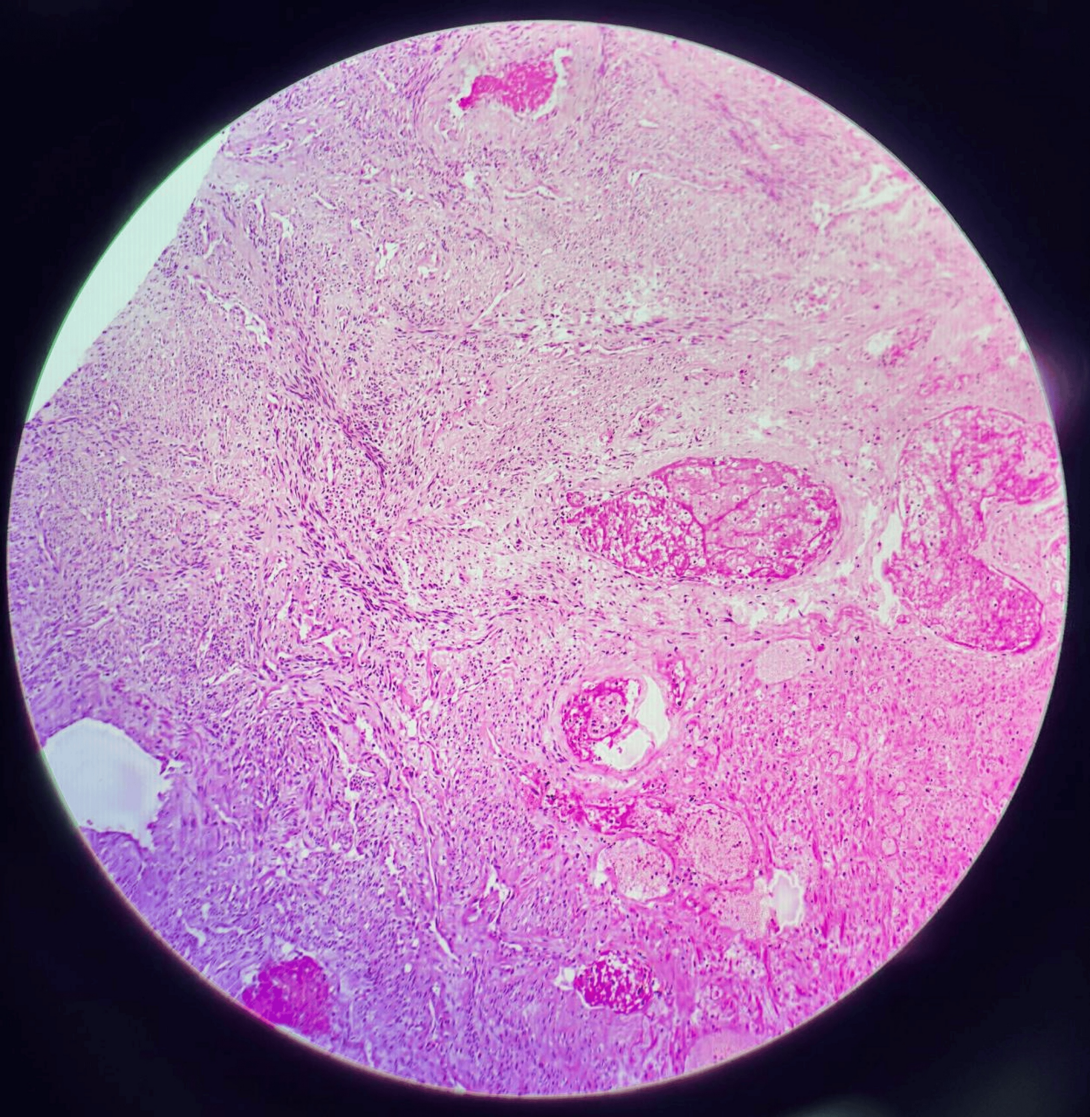
Case 1: Histopathology, haematoxylin and eosin stain, and leiomyoma with hydropic changes and areas of necrosis Photomicrograph (haematoxylin and eosin, low power) demonstrating interlacing bundles of bland smooth muscle cells arranged in a whorled pattern with prominent hydropic degeneration evident as pale oedematous areas within and between the smooth muscle fascicles. Dilated thin-walled vascular channels are identified. Areas of necrosis are also present. No significant nuclear atypia or increased mitotic activity was observed, consistent with degenerative changes in a benign leiomyoma rather than malignant necrosis, thereby excluding the preoperative MRI concern for leiomyosarcoma.

The histopathological findings on haematoxylin and eosin staining, confirming leiomyoma with hydropic changes and areas of necrosis, are illustrated in Figure 3. The patient made an uncomplicated recovery and was discharged on the eighth postoperative day.

Case 2

Cervical Fibroid With Multiple Degenerative Fibroids and Bilateral Ureteric Dilatation: Three-Dimensional CT Mapping and Bilateral DJ Stenting

A 59-year-old woman (P2L2A1) with type 2 diabetes mellitus presented with right flank pain for eight days, radiating to the back and worsening during menses. MRI pelvis revealed a bulky uterus (8.7 × 10.5 × 11.9 cm) with multiple intramural and subserosal fibroids (International Federation of Gynecology and Obstetrics (FIGO) types 3-6), the largest subserosal fibroid measuring 5.8 × 8.8 × 5.7 cm.

Due to fibroid complexity and limited ureteric visualisation on MRI, abdominal/pelvic contrast-enhanced CT (CECT) with 3D volume rendering was performed, revealing a bulky uterus (~12 × 7.1 × 6.9 cm) with multiple well-defined, heterogeneously hypoattenuating fibroids. The dominant and most surgically significant lesion was a large lower uterine segment and anterior cervical fibroid measuring 11 × 5.7 × 7.2 cm, situated at the most anatomically hazardous location for ureteric injury, where the ureter crosses beneath the uterine artery. Additional fibroids included a subserosal fibroid (FIGO 6; 4.2 × 4.8 × 6.3 cm, posterior fundus) and multiple subserosal fibroids (FIGO 5, right anterolateral wall) with coarse calcifications.

Critically, the CECT demonstrated bilateral ureteric dilatation due to mass effect: the right ureter coursed in close proximity to the right lateral uterine surface with mild dilatation throughout its course, while the left ureter was in close proximity to the left lateral uterine surface with dilatation of the proximal and mid ureter. Prominent bilateral pelvicalyceal systems were noted. The three-dimensional volume rendering provided a vivid visual representation of bilateral ureteric displacement in relation to the fibroid mass.

**Figure 4:**
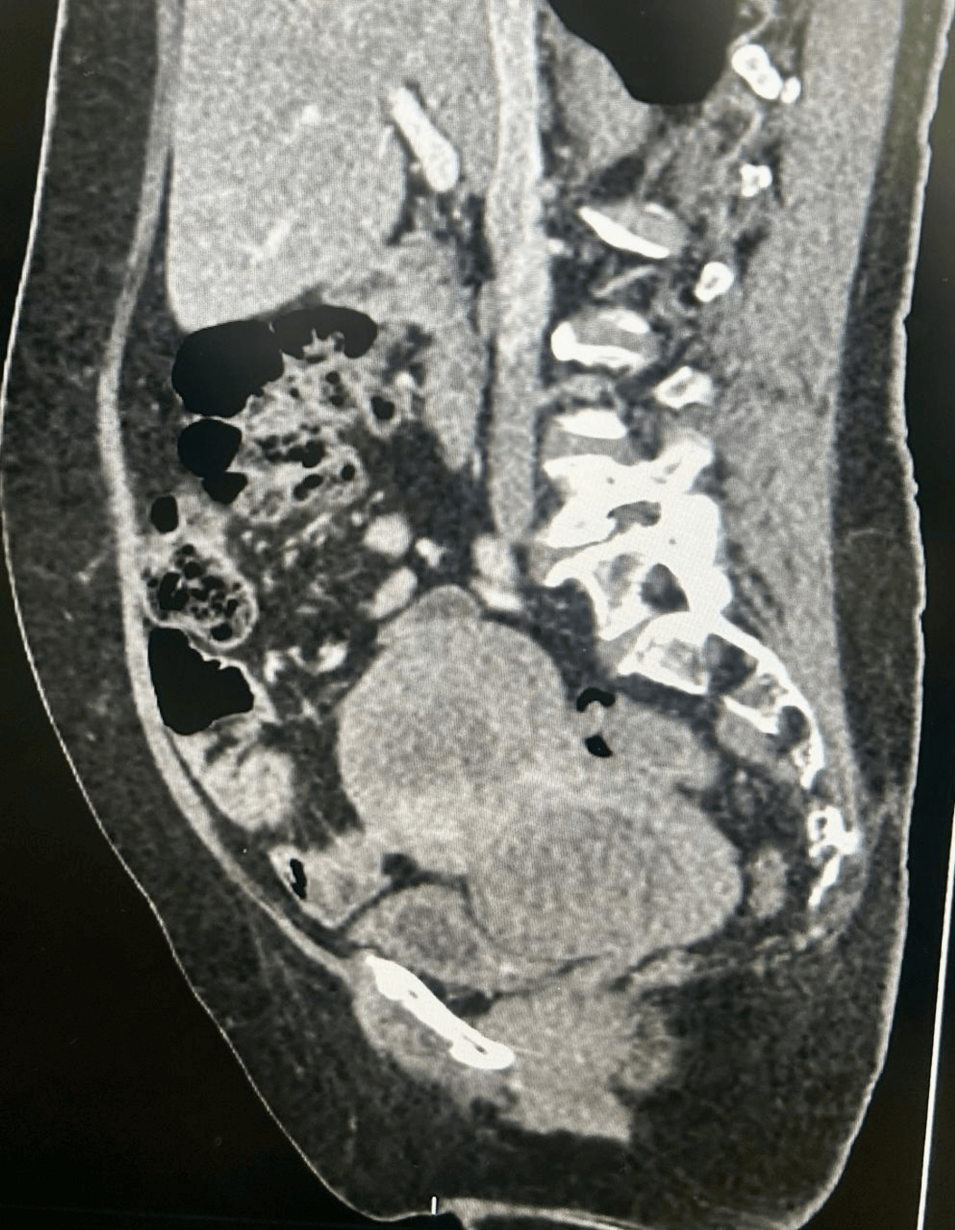
Case 2: Sagittal reconstruction of contrast-enhanced CT of the abdomen and pelvis Sagittal contrast-enhanced CT demonstrating the dominant lower uterine segment and cervical fibroid as a large hypodense pelvic mass, with the lumbar spine visible posteriorly. The inferior extent of the mass and its proximity to the bladder and pelvic floor are clearly delineated, illustrating the anatomical complexity and the risk of ureteric displacement at the cardinal ligament level.

**Figure 5:**
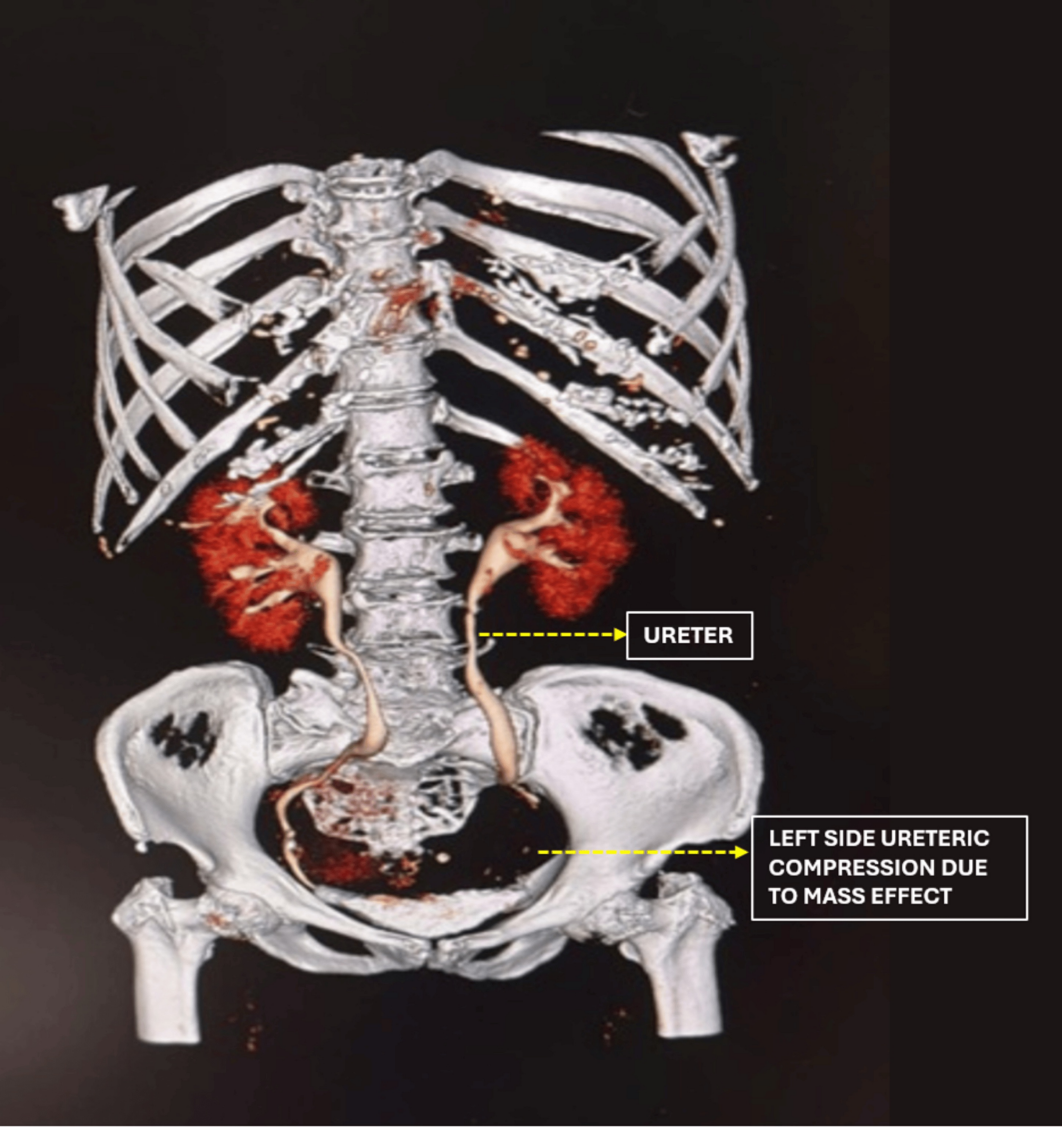
Case 2: Three-dimensional CT volume rendering of contrast-enhanced CT of the abdomen and pelvis Bilateral ureteric displacement is clearly demonstrated as both ureters course alongside the enlarged fibroid uterus before entering the pelvis. The dominant lower uterine segment and cervical fibroid (11 × 5.7 × 7.2 cm) causes medial displacement and dilatation of both ureters. This three-dimensional reconstruction formed the basis for bilateral prophylactic DJ stenting. DJ: Double-J.

The sagittal contrast-enhanced CT reconstruction demonstrating the dominant lower uterine segment and cervical fibroid is shown in Figure 4, and the three-dimensional CT volume rendering illustrating bilateral ureteric displacement is shown in Figure 5.

This case presented a dual intraoperative challenge: the large cervical fibroid at the lower uterine segment and bilateral ureteric dilatation from mass effect. In view of both these findings, bilateral prophylactic DJ stenting was performed preoperatively under cystoscopic guidance.

**Figure 6:**
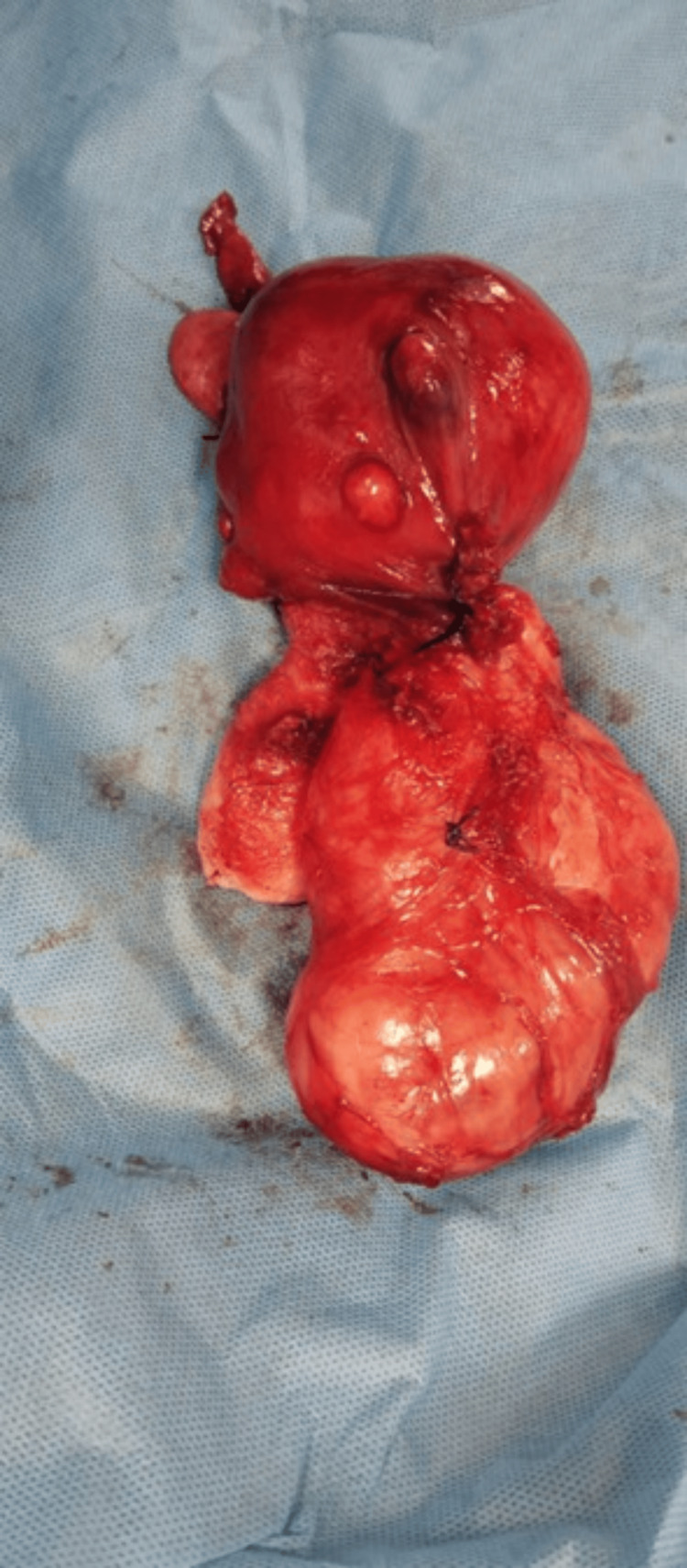
Case 2: Gross specimen photograph of the uterus with fibroids following total hysterectomy and bilateral salpingo-oophorectomy Gross specimen following total abdominal hysterectomy and bilateral salpingo-oophorectomy, demonstrating the enlarged uterus with multiple fibroids of varying sizes. The dominant lower uterine segment and cervical fibroid are clearly identified.

**Figure 7:**
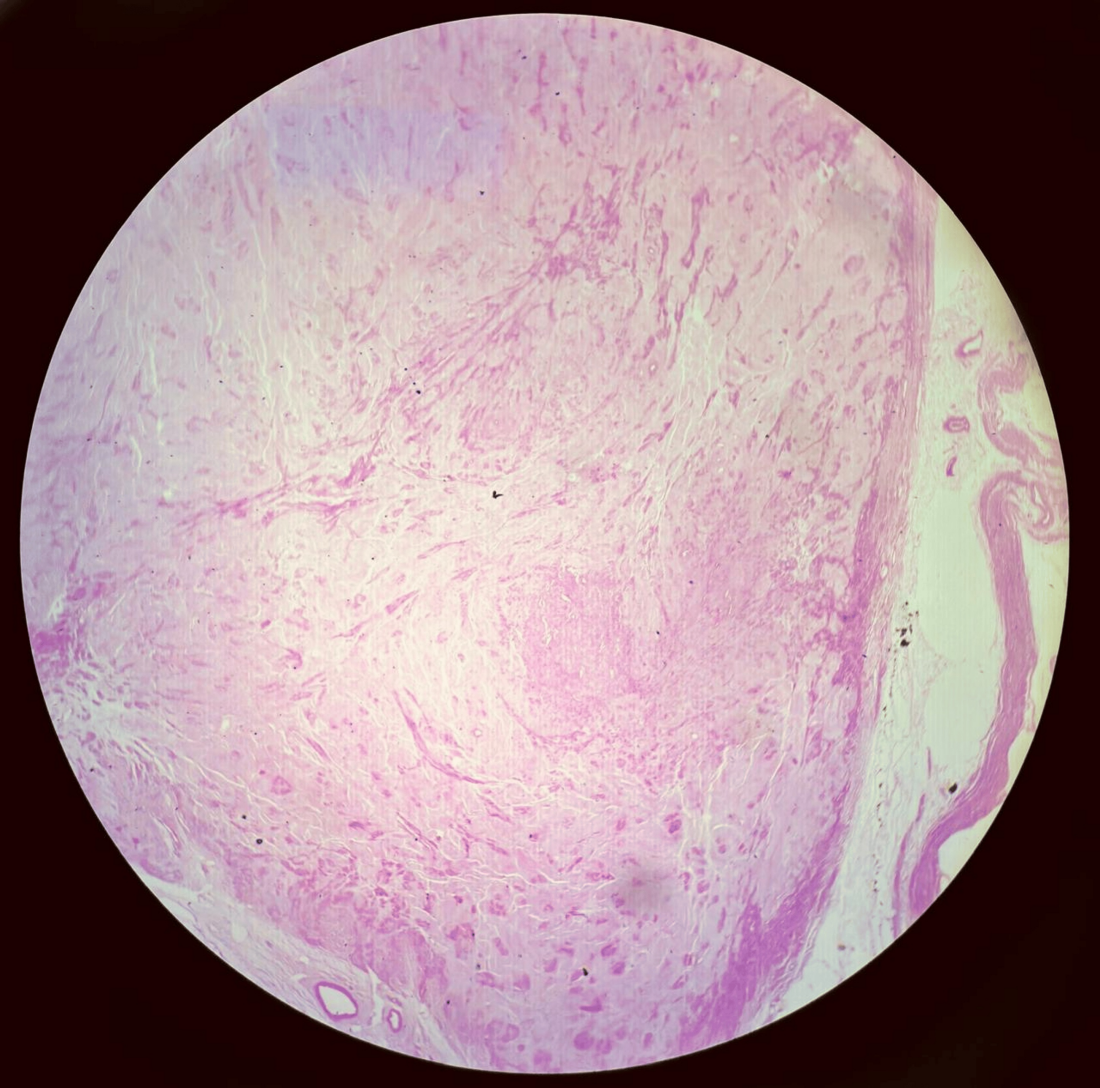
Case 2: Histopathology, haematoxylin and eosin stain, and leiomyoma with hyaline degeneration Photomicrograph (haematoxylin and eosin, low power) demonstrating smooth muscle bundles with extensive hyaline degeneration, evidenced by large acellular pale eosinophilic zones replacing the tumour parenchyma. The residual smooth muscle fascicles show no significant nuclear atypia or mitotic activity.

Total abdominal hysterectomy with bilateral salpingo-oophorectomy was performed. Bilateral retroperitoneal dissection was systematically performed, with the ureteric course on both sides confirmed and traced in conjunction with the pre-placed stents. The cervical fibroid required careful dissection of the bladder and paracervical tissue, with continuous ureteric visualisation maintained throughout. No ureteric injury occurred. The DJ stents were removed postoperatively. The gross specimen following total abdominal hysterectomy and bilateral salpingo-oophorectomy is shown in Figure 6, and the histopathological findings confirming leiomyoma with hyaline degeneration are illustrated in Figure 7. The patient was discharged without complication.

Case 3

Broad Ligament Fibroid With Flank Pain: Enucleation of the Fibroid Within the Intracapsular Plane Before Uterine Artery Ligation

**Figure 8:**
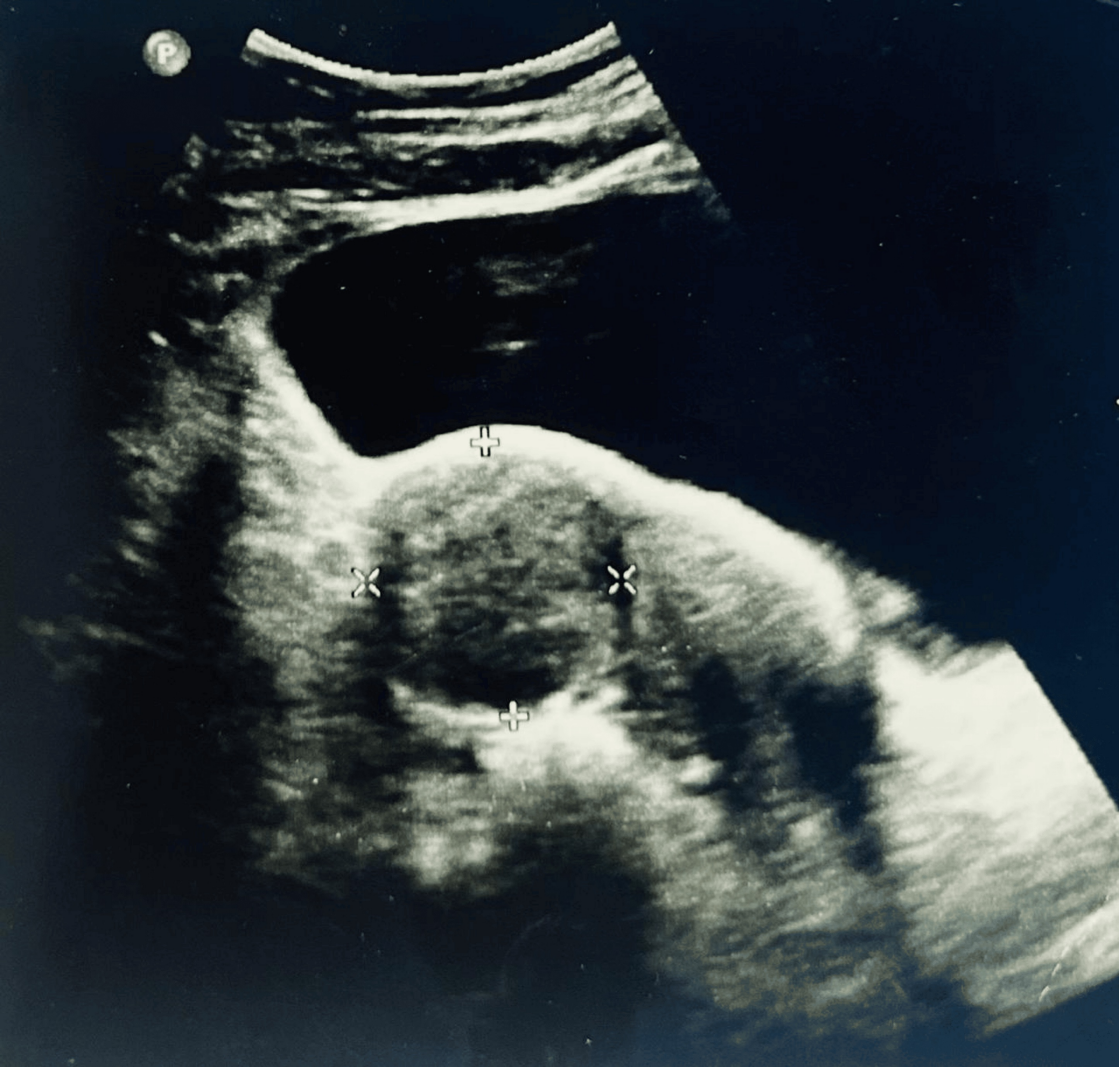
Case 3: Ultrasonography of the abdomen and pelvis Ultrasonography demonstrating a large heterogeneous pelvic mass with measurement callipers, consistent with a broad ligament fibroid adjacent to the right lateral uterine wall. The lesion measures approximately 4.1 × 3.8 cm, with the uterus deviated to the left. Despite the absence of overt hydronephrosis, the proximity of the mass to the right ureter prompted heightened intraoperative vigilance and systematic retroperitoneal dissection for ureteric identification during the planned procedure.

A 45-year-old woman (P2L2A1) with type two diabetes mellitus presented with right-sided flank pain of eight days' duration, radiating to the back and aggravated at the time of menstruation. Ultrasonography demonstrated a well-defined hypoechoic solid lesion measuring 4.1 × 3.8 cm adjacent to the right lateral uterine wall, suggestive of a subserosal or broad ligament fibroid (Figure 8), along with additional multiple subserosal fibroids (largest 4.15 × 4.17 cm on left lateral wall). A left renal calculus of 0.5 cm was noted without hydronephrosis.

On clinical examination, the uterus was anteverted, deviated to the left, 12-14 weeks in size, irregular, and mobile. The right-sided flank pain was clinically attributed to ureteric irritation or compression from the adjacent broad ligament fibroid, even in the absence of overt hydronephrosis on imaging - a finding that reinforced the importance of vigilance regarding ureteric proximity during the planned procedure.

**Figure 9:**
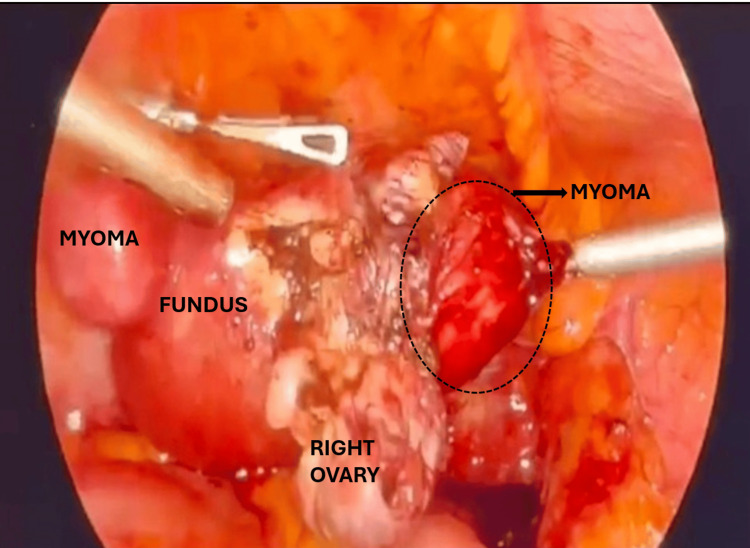
Case 3: Laparoscopic intraoperative photograph of the broad ligament fibroid held with myoma screw Laparoscopic intraoperative view demonstrating the broad ligament fibroid secured with a myoma screw for traction during a laparoscopic hysterectomy with bilateral salpingectomy. Controlled traction on the fibroid facilitated careful dissection of the surrounding tissue planes. Systematic retroperitoneal identification and tracing of the right ureter were performed prior to this step to ensure ureteric safety throughout the dissection. No ureteric injury occurred.

**Figure 10:**
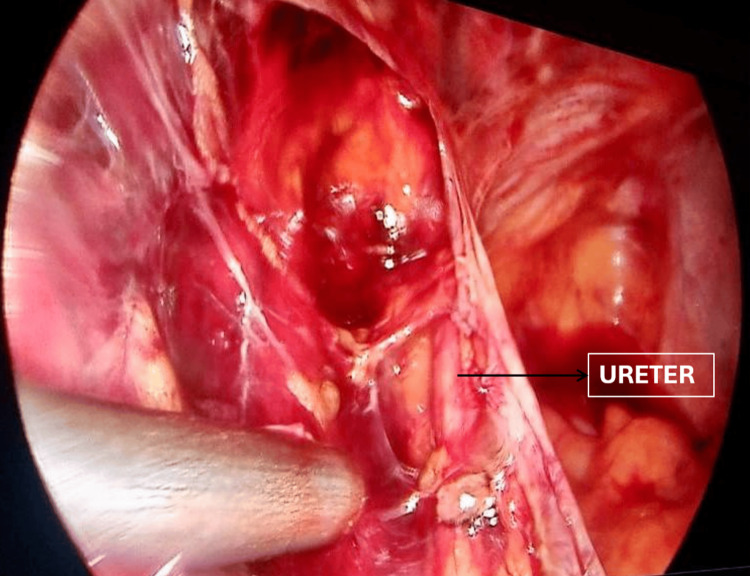
Case 3: Laparoscopic intraoperative photograph demonstrating retroperitoneal identification of the right ureter Laparoscopic intraoperative view demonstrating the right ureter clearly identified in the retroperitoneal space following systematic dissection through the avascular plane between the infundibulopelvic ligament and the round ligament. The ureter is indicated by the arrow. Direct visualisation of the ureteric course was maintained throughout the dissection, precluding ureteric injury during laparoscopic hysterectomy with bilateral salpingectomy for broad ligament fibroid.

The patient underwent a laparoscopic hysterectomy with bilateral salpingectomy. The primary intraoperative ureteric safeguarding strategy was enucleation of the fibroid within the intracapsular plane, systematic retroperitoneal dissection with anatomical identification of the right ureter at the pelvic brim, followed by careful tracing of its course away from the fibroid mass before proceeding with dissection. No prophylactic DJ stent was inserted. No ureteric injury occurred. The laparoscopic intraoperative photograph of the broad ligament fibroid held with a myoma screw is shown in Figure 9, and retroperitoneal identification of the right ureter following systematic dissection is demonstrated in Figure 10.

**Figure 11:**
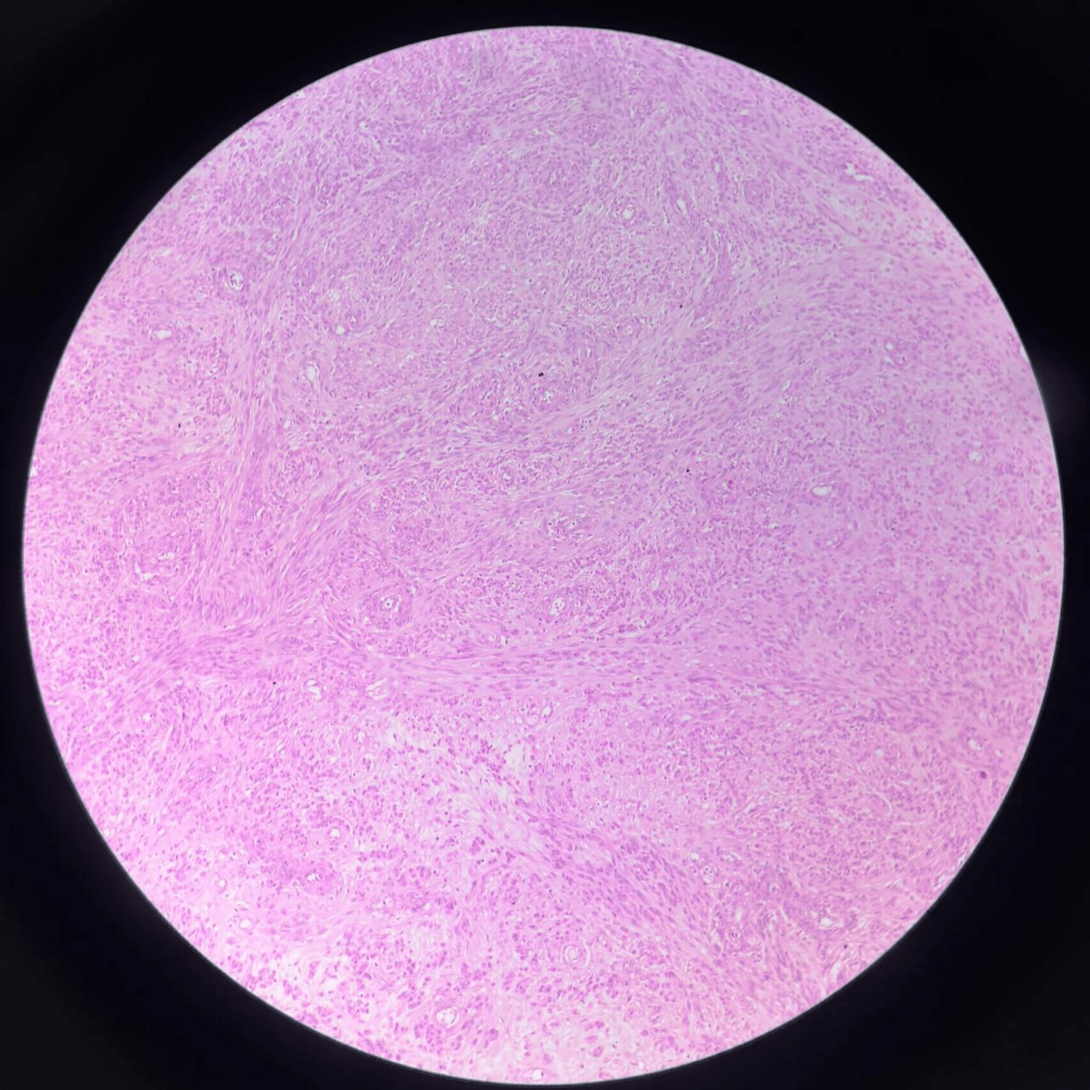
Case 3: Histopathology, haematoxylin and eosin stain, and leiomyoma Photomicrograph (haematoxylin and eosin, low power) demonstrating a cellular tumour composed of interlacing bundles of bland spindle-shaped smooth muscle cells arranged in a whorled and fascicular pattern. The smooth muscle cells display uniform, elongated nuclei with no significant pleomorphism, hyperchromasia, or increased mitotic activity.

The histopathological findings on haematoxylin and eosin staining are shown in Figure 11. The patient was discharged without complication.

Case 4

Pseudo-Broad Ligament Fibroids With Failed Left-Sided DJ Stenting: Retroperitoneal Dissection as Salvage Strategy

A 46-year-old woman (P2L2) presented with irregular menses and heavy menstrual bleeding of two months' duration. CT confirmed a 9 × 10 × 5 cm ill-defined lesion in the left adnexa with right uterine displacement, suggestive of a broad ligament or large subserosal fibroid. On clinical examination, a hypogastric mass of 14-16 weeks' size was palpated, with the uterus filling the left side of the pelvis and palpable in the pouch of Douglas.

Given the size and location of the left-sided mass and its proximity to the left ureter, preoperative bilateral DJ stenting was planned. Under cystoscopic guidance, the right-sided stent was inserted without difficulty. However, during attempted left-sided stenting, obstruction was encountered, and the guidewire could not be advanced - almost certainly reflecting significant left ureteric compression or displacement by the broad ligament fibroid mass. Left-sided stenting was abandoned after repeated failed attempts. This finding was itself of diagnostic and prognostic significance, as the inability to stent the left ureter confirmed substantial ureteric compromise and informed the intraoperative team of the need for meticulous retroperitoneal dissection on the left side.

**Figure 12:**
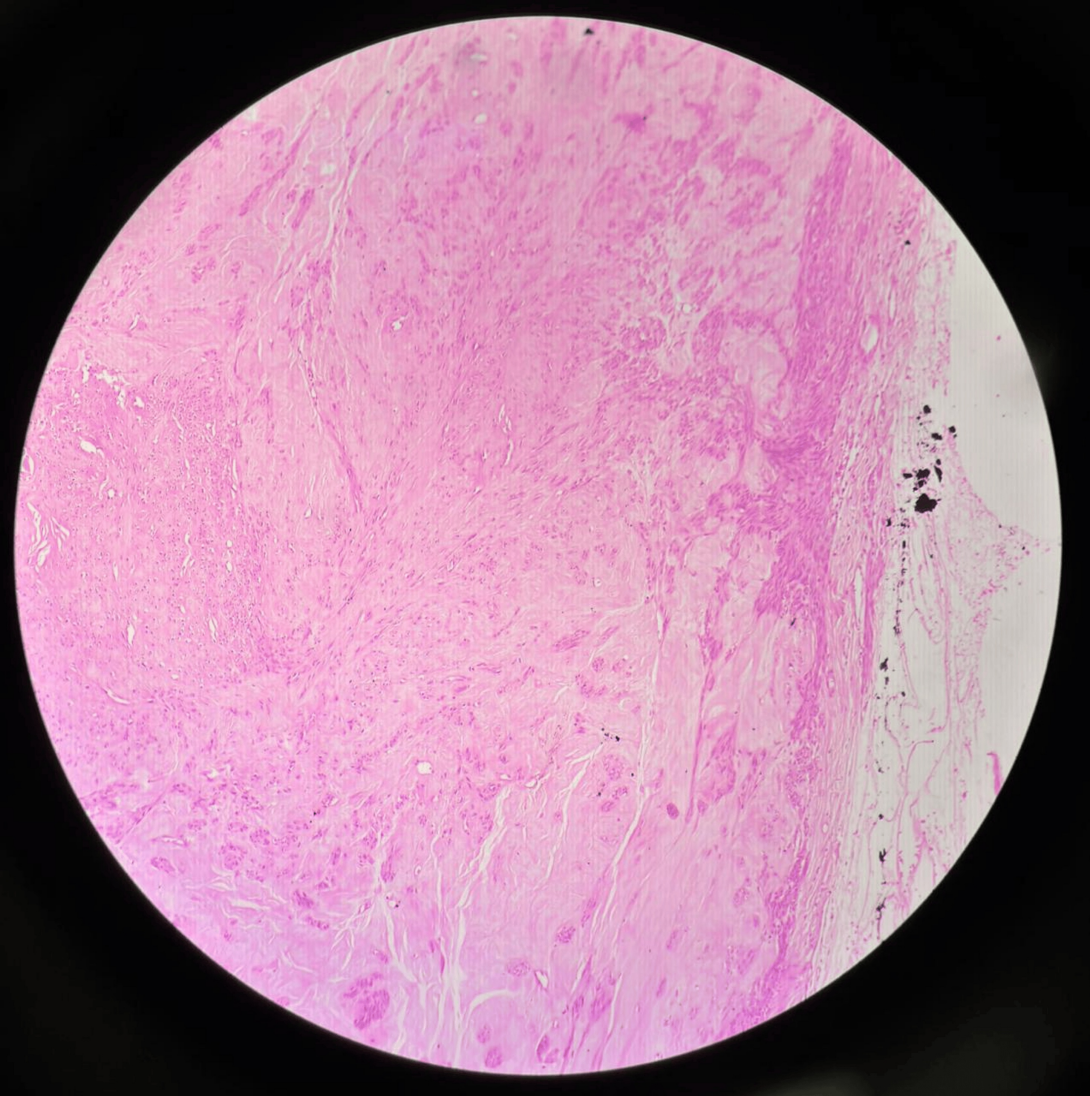
Case 4: Histopathology, haematoxylin and eosin stain, and leiomyoma with myxoid and hyaline degeneration Photomicrograph (haematoxylin and eosin, low power) demonstrating smooth muscle bundles with prominent myxoid and hyaline degenerative changes. The tumour shows interlacing fascicles of bland smooth muscle cells set against a pale myxoid stroma, with acellular hyalinised zones interspersed throughout. No significant nuclear pleomorphism, increased mitotic activity, or coagulative tumour cell necrosis is identified. These features are consistent with a benign leiomyoma with degenerative changes, correlating with the preoperative imaging appearances of the pseudo-broad ligament fibroid.

Total abdominal hysterectomy with left salpingo-oophorectomy and right salpingectomy was performed. Intraoperative findings revealed two large pseudo-broad ligament fibroids arising from the uterine isthmus: the larger fibroid (10 × 7 cm) was situated deep in the left retroperitoneal space, and a smaller fibroid (5 × 5 cm) was situated anterior to the uterus and pushing the bladder upwards. A 5 × 5 cm subserosal fibroid was also identified arising from the right uterine body and cornual area. The retroperitoneum was entered through the space between the left infundibulopelvic ligament and the left round ligament. The course of the left ureter was systematically delineated, and the pseudo-broad ligament fibroid was carefully dissected out under direct ureteric vision. The bilateral uterine arteries were identified and clamped after confirming the ureteric position. No ureteric injury occurred. The right DJ stent was removed postoperatively. The patient was discharged on postoperative day 5 without complications. Histopathological examination (Figure 12) revealed​ a​​​​​ leiomyoma with myxoid and hyaline degeneration.

The CT image for Case 4 is not available, as the original file was lost due to data corruption and could not be retrieved.

Case 5

Large Left Broad Ligament Fibroid: Intraoperative Ureteric Transection, Immediate Recognition, and Successful Repair

**Figure 13:**
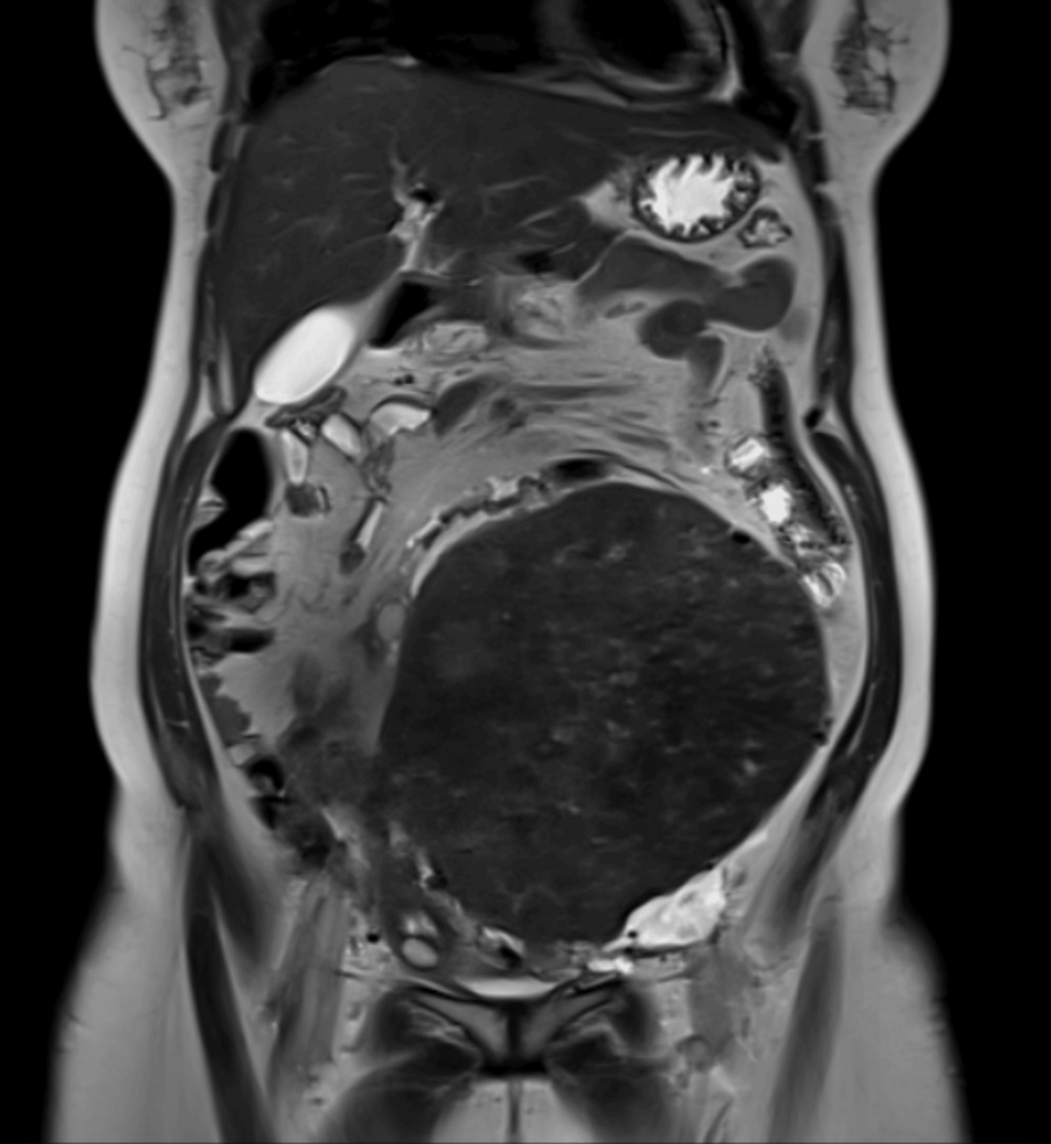
Case 5: MRI of the abdomen and pelvis, axial T2-weighted sequence Axial T2-weighted MRI demonstrating the large, well-defined ovoid mass measuring 21 × 13 × 17 cm in the left adnexal region, occupying the pelvis and displacing the uterus and bowel superiorly and to the right. The mass demonstrates heterogeneous signal intensity with peripheral cystic areas, consistent with the ovarian pedicle sign identified on imaging.

A 47-year-old woman (P2L2, prior two lower segment caesarean sections and incisional hernia repair) presented with progressive abdominal distension for three years and abnormal uterine bleeding. CT scan demonstrated an approximately 15 × 15 cm ill-defined, heterogeneously enhancing solid pelvic mass reaching the anterior abdominal wall, suggestive of a broad ligament fibroid or adnexal lesion. MRI abdomen and pelvis confirmed a large ovoid, well-delineated enhancing mass measuring 21 × 13 × 17 cm in the left adnexal region with ovarian pedicle sign, peripheral cystic areas, and displacement of adjacent structures without evident infiltration (Figure 13). A subserosal uterine fibroid measuring 2.5 × 2.7 cm was noted anteriorly, and the uterus was bulky and globular with changes suggestive of adenomyosis. CA-125 was within normal limits at 21.2 IU/mL, although serum lactate dehydrogenase (LDH) was elevated at 330 IU/L, maintaining a degree of preoperative oncological vigilance. On clinical examination, the uterine mass was 24-26 weeks in size, smooth, irregular, mobile, and deviated to the left.

At total abdominal hysterectomy with bilateral salpingo-oophorectomy, intraoperative findings revealed the uterus to be approximately 12 weeks in size. A large fibroid arising from the left side of the uterus measuring 30 × 20 × 15 cm was identified with prominent vascularity in the left lateral wall. The round ligament was stretched and displaced; its outline was carefully traced, identified, clamped, and cut. The peritoneum overlying the mass was dissected carefully. Posterior adhesions to the bowel were released by sharp dissection. The infundibulopelvic ligament was skeletonised and ligated under direct vision. During bladder mobilisation and haemostasis of vascular channels posterior to the lateral wall peritoneum, inadvertent left ureteric transection occurred beneath a vascular clamp.

The ureteric transection in this case represented a Grade IV injury according to the American Association for the Surgery of Trauma (AAST) Organ Injury Scale, involving complete transection with less than 2 cm of devascularisation - a severity that mandates immediate surgical reconstruction rather than conservative management [6].

The injury was recognised immediately. Both the proximal and distal ends of the transected left ureter were identified, with clear urine noted from the proximal end. Vascularity was found intact at both ends. A titanium guidewire was inserted into the proximal ureteric end to a depth of 20 cm. An open-ended DJ stent was inserted onto the guidewire; the guidewire was then removed, and the distal ureteric end was directly intubated successfully. The transected ends were anastomosed using 4-0 polydioxanone (PDS) interrupted sutures, with knots placed on the extraluminal side (ureteroureterostomy). No urinary leakage was identified on testing.

**Figure 14:**
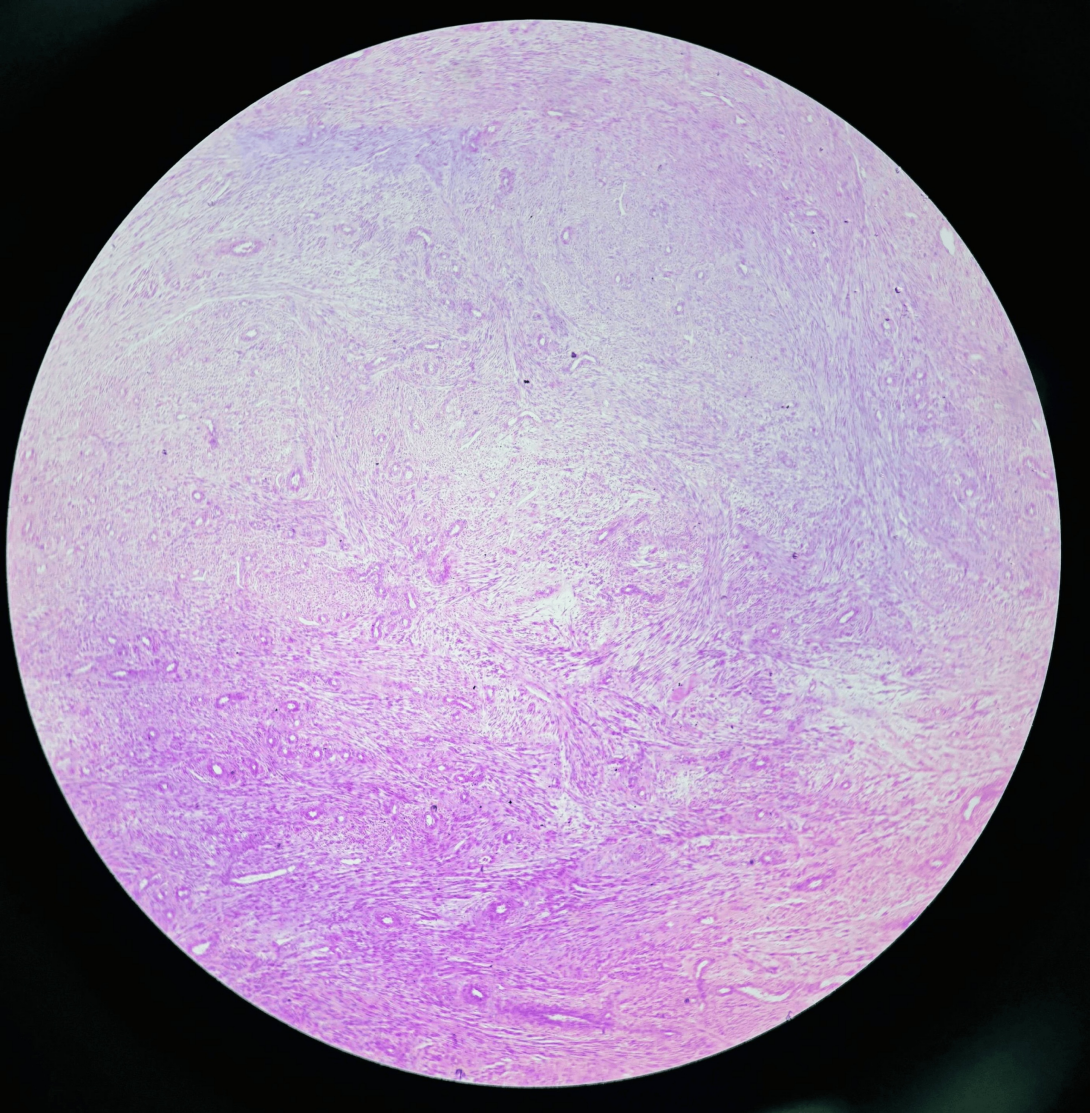
Case 5: Histopathology, haematoxylin and eosin stain, and leiomyoma Photomicrograph (haematoxylin and eosin, low power) demonstrating interlacing bundles of bland smooth muscle cells with prominent oedematous and hyaline degenerative changes. The smooth muscle fascicles are separated by pale oedematous stroma with acellular hyalinised zones. No significant nuclear pleomorphism, increased mitotic activity, or coagulative tumour cell necrosis is identified.

A silicone urethral catheter was maintained for seven days. The abdominal drain was removed on postoperative day 7. The patient was discharged in stable condition and was advised to follow up with urology and undergo cystoscopy at 21 days postoperatively. The DJ stent was removed postoperatively as planned. The patient made an uncomplicated recovery. The histopathological findings on haematoxylin and eosin staining confirmed leiomyoma, as illustrated in Figure 14.

The clinical features, imaging modalities, ureteric safeguarding strategies, and outcomes across all five cases are summarised in Table 1.

**Table 1:** Summary of clinical features, imaging, ureteric strategies, and outcomes across all five cases BL: Broad ligament; DJ: Double-J; HPE: Histopathological examination; CECT: Contrast-enhanced CT.

Feature	Case 1	Case 2	Case 3	Case 4	Case 5
Age (years)	33	59	45	46	47
Parity	P2L2A1	P2L2	P2L2A1	P2L2	P2L2
Preoperative diagnosis	Giant broad ligament/subserosal fibroid	Cervical fibroid + multiple degenerative fibroids + bilateral ureteric dilatation	Broad ligament fibroid + multiple subserosal fibroids	Left pseudo-broad ligament fibroids (×2) + right subserosal fibroid	Large left broad ligament fibroid + adenomyosis
Fibroid size	30 × 24 × 20 cm (bilobed)	Cervical fibroid 11 × 5.7 × 7.2 cm (dominant); subserosal 4.2 × 4.8 × 6.3 cm; multiple others	4.1 × 3.8 cm (index fibroid)	10 × 7 cm + 5 × 5 cm (pseudo-BL)	30 × 20 × 15 cm
Imaging	MRI (fibroid mapping)	MRI + CECT + 3D ureteric mapping	Ultrasonography	Ultrasonography + CT scan	CT + MRI
Preop DJ stent	No	Yes (bilateral)	No	Right: Yes; Left: Failed	Not attempted preoperatively
Ureteric strategy	Retroperitoneal dissection + anatomical identification	Three-dimensional mapping + bilateral DJ stenting	Enucleation of fibroid prior to uterine artery ligation	Right side DJ stenting + left side meticulous ureteric tracing in view of failed stenting	Timely intraoperative recognition + preparedness in view of dense adhesion + ureteroureterostomy
Procedure	Open myomectomy + frozen section	Total abdominal hysterectomy + bilateral salpingo-oophorectomy	Laparoscopic hysterectomy + bilateral salpingectomy	Total abdominal hysterectomy + left salpingo-oophorectomy + right salpingectomy	Total abdominal hysterectomy + bilateral salpingo-oophorectomy + left ureteric transection repair
HPE	Leiomyoma with necrosis and hydropic changes	Leiomyoma with hyaline degeneration	Leiomyoma	Leiomyoma with myxoid and hyaline degeneration	Leiomyoma
Ureteric outcome	No injury	No injury	No injury	No injury	Left ureteric transection: recognised intraoperatively, repaired successfully

## Discussion

This case series illustrates the challenges of operating on large fibroids in close proximity to the ureter and proposes a multimodal safeguarding framework. Taken together, our five cases offer a framework that progresses from preoperative anticipation to intraoperative strategy and, when necessary, damage control, reflecting the full reality of complex gynaecological surgery. Ureteric injury during gynaecological surgery occurs at predictable anatomical locations. The ureter is most vulnerable at the infundibulopelvic ligament during ligation of the ovarian vessels, at the uterine artery crossing where it passes beneath the artery (‘water under the bridge’), and at the ureterovesical junction during bladder dissection and vaginal cuff closure [12]. Understanding these zones informs both the preoperative imaging strategy and the intraoperative dissection plan.

The reported incidence of ureteric injury varies considerably across procedure types and surgical complexity, as summarised in Tables 2, 3. Vaginal hysterectomy carries the lowest risk at 0.02%-0.06%, whereas radical gynaecological oncology surgery carries the highest rates, reaching 10.7% in large national cohort data [2-5,7,8]. This wide variation underscores the importance of individualised preoperative risk stratification rather than a uniform approach.

**Table 2:** Ureteric injury rates in gynaecological surgeries from selected studies Data compiled from published literature. This table was created by the authors. Hx: Hysterectomy; LAVH: Laparoscopic-assisted vaginal hysterectomy; OR: Odds ratio; CI: Confidence interval; lap: Laparoscopic; NHS: National Health Service.

Study/Reference	Surgery Type	No. of Cases	Ureteric Injury Rate	Notes
Brummer et al., 2011 [13]	Abdominal hysterectomy	5,279	0.30%	Prospective nationwide Finnish cohort (FINHYST)
	Vaginal hysterectomy	5,279	0.04%	Lowest ureteric injury rate across approaches
	Laparoscopic hysterectomy	5,279	0.30%	Similar risk to the abdominal approach
Wei et al., 2023 [14]	Vaginal hysterectomy	—	0.01%	Weighted pooled mean; 96 studies; 1.74 M women
	Laparoscopic Hx (benign)	—	0.26%	Higher rate than the vaginal approach
	Laparoscopic Hx (malignancy)	—	0.81%	Elevated risk in oncologic setting
	LAVH	—	0.20%	Intermediate risk; pooled estimate
Adelman et al., 2014 [15]	Laparoscopic hysterectomy	—	0.02%-0.4%	Systematic review; 40 studies; PubMed 10 years
Soong et al., 2007 [16]	LAVH	>1,000	0.10%	Prospective series; urinary tract injury focus
Wong et al., 2018 [8]	Endometriosis surgery (lap)	—	≈0.4%	Highest ureteric risk among benign indications
Yanagisawa et al., 2023 [7]	Laparoscopic vs open Hx	—	OR 2.12 (CI 1.71-2.62)	Meta-analysis; 46 studies; lap associated with higher risk
Kiran et al., 2016 [17]	Radical Hx (uterine cancer)	3,77,073	10.70%	10-year NHS cohort; highest rate in radical abdominal Hx
	All hysterectomies (benign)	—	<1%	Typical range for non-oncologic surgery

**Table 3:** Simplified comparative risk across common gynaecological procedures Data compiled from published literature. This table was created by the authors. LAVH: Laparoscopic-assisted vaginal hysterectomy.

Procedure	Approximate Risk of Ureteric Injury	Source
Vaginal hysterectomy	0.02%-0.06%	Brummer et al., 2011 [13]; Wei et al., 2023 [14]
Abdominal (open) hysterectomy	0.1%-0.3%	Brummer et al., 2011 [13]; Kiran et al., 2016 [17]
Laparoscopic hysterectomy (benign)	0.02%-0.4%	Adelman et al., 2014 [15]; Yanagisawa et al., 2023 [7]
LAVH	0.08%-0.2%	Soong et al., 2007 [16]; Wei et al., 2023 [14]
Endometriosis surgery	≈0.4%	Wong et al., 2018 [8]
Radical gynaecologic oncology surgery	1%-10.7%	Yanagisawa et al., 2023 [7]; Kiran et al., 2016 [17]

The role of preoperative imaging and ureteric mapping

Preoperative imaging plays a pivotal role in individualised management. Fibroid mapping through MRI assists in visualisation of the fibroid's relationship to the lateral pelvic sidewall, enabling anticipatory retroperitoneal planning.

The CECT with three-dimensional reconstruction can provide an intuitive map in cases of cervical and lower segment large fibroids. The three-dimensional volume rendering can provide a projection of bilateral ureteric displacement [2,3].

In Cases 3 and 4, ultrasonography and CT, respectively, provided sufficient anatomical delineation to plan the ureteric strategy, underscoring that the choice of imaging modality should be tailored to the complexity of the individual case rather than applied uniformly.

Prophylactic DJ ureteric stenting: utility and limitations

The role of prophylactic ureteric stenting in gynaecological surgery remains debated in the literature. Proponents argue that stenting facilitates intraoperative ureteric identification, particularly in cases of distorted anatomy, and enables earlier recognition of injury. Critics note that stenting is not without its own complications - including ureteric trauma during insertion, urinary tract infection, stent migration, and patient discomfort - and that it does not prevent all injuries [4].

Our series adds nuance to this debate. In Case 2, bilateral preoperative stenting was performed successfully and contributed to ureteric safety during dissection in a case with documented bilateral ureteric dilatation. In Case 4, however, the attempted left-sided stenting failed due to fibroid-related obstruction - a finding that paradoxically provided highly valuable clinical information. The inability to pass the stent confirmed significant left ureteric compression or angulation, directly alerting the surgical team to the degree of ureteric compromise and the need for especially meticulous retroperitoneal dissection on the left side. This case illustrates that a failed stenting attempt should not be dismissed as a procedural failure but rather interpreted as a diagnostic signal in its own right.

In Cases 1 and 3, preoperative stenting was not performed, and ureteric safety was achieved through retroperitoneal dissection and anatomical identification alone, reinforcing that stenting is a complementary rather than mandatory component of the protocol and should be applied selectively based on preoperative risk stratification.

Intraoperative considerations

From the perspective of fibroid-specific surgical planning, true broad ligament and cervical fibroids warrant particular attention. Preoperative three-dimensional MRI fibroid mapping aids in delineating the ureteric course and planning the line of dissection, with the primary operative principle being enucleation of the fibroid within the intracapsular plane [12]. In the majority of cases, the ureter lies closely applied within the overstretched layers of the broad ligament and must be actively identified rather than assumed. In bulky central cervical fibroids, significant cranial migration of the ureters occurs bilaterally due to expansion of the isthmic region, making ligation at the level of the uterine artery the most vulnerable step. A protective strategy in such cases is to make a midline vertical incision over the central fibroid posteriorly and proceed with myomectomy before the standard steps of hysterectomy, thereby creating adequate operative space for safe ureteric identification [1].

Retroperitoneal dissection as the cornerstone of ureteric safety

Across all five cases, systematic retroperitoneal dissection was the consistent intraoperative strategy. Entry into the retroperitoneal space through the avascular plane between the infundibulopelvic ligament and the round ligament allows identification of the ureter at the pelvic brim, from which it can be traced distally under direct vision. This approach converts a potentially dangerous blind dissection into a controlled, anatomy-guided procedure [18].

Procedure-specific intraoperative considerations

Beyond the fibroid cases presented in this series, the principles of ureteric safeguarding are applicable across a broad spectrum of gynaecological procedures, each of which carries distinct anatomical risks.

In surgery for endometriosis, dense adhesions, fibrosis, radical hysterectomy, and obliteration of normal tissue planes substantially elevate the risk of ureteric injury. Early retroperitoneal entry with systematic ureteric identification and tracing is strongly recommended [1,12]. Where visualisation remains inadequate, ureteric cannulation with instillation of indocyanine green dye provides real-time delineation of the ureteric course under fluorescence guidance, permitting safer directed dissection. Sharp dissection with minimal use of thermal energy should be employed in proximity to the ureter, as electrosurgical injuries may not be apparent intraoperatively and characteristically present in the postoperative period with obstruction or fistula formation. In cases of suspected ureteric involvement, proactive ureterolysis and selective stenting may facilitate safer tissue planes [1,12].

In abdominal hysterectomy, both open and laparoscopic, the principle of meticulous skeletonisation of the uterine arteries and veins is paramount, ensuring that vessel ligation is precise and anatomically defined. In laparoscopic hysterectomy specifically, liberal use of energy devices in the absence of confirmed ureteric position should be avoided. When intraoperative bleeding obscures the operative field, using small pressure swabs and allowing the coagulation cascade to achieve haemostasis are preferable to indiscriminate application of energy sources, which carries a significant risk of occult thermal ureteric injury [12,18-20].

In vaginal hysterectomy, although the overall risk of ureteric injury is lower, injury may occur during clamping of the uterosacral or cardinal ligaments. Clamps should be placed close to the cervix, and excessive lateral dissection should be avoided. Adequate bladder mobilisation and continuous awareness of ureteric proximity during vault closure are essential preventive steps [12].

Adjunctive intraoperative verification and postoperative monitoring

In high-risk cases, adjunctive intraoperative techniques, including cystoscopy with confirmation of bilateral ureteric jets and instillation of methylene blue or indigo carmine dye, provide a valuable additional layer of injury detection at the conclusion of the procedure [8,9,20]. Ureteric injuries - particularly thermal or ischaemic injuries - may present in a delayed fashion with non-specific features including flank pain, fever, unexplained ileus, reduced urine output, or rising serum creatinine. A high index of clinical suspicion in the postoperative period, particularly following high-risk procedures, enables early investigation with renal ultrasonography or CT urography, facilitating timely diagnosis and maximising the opportunity for renal salvage [1,20].

Case 5: the importance of immediate recognition and definitive intraoperative repair

Case 5 is the most instructive in this series, precisely because injury occurred despite thorough preoperative risk assessment, surgical awareness, and preparedness. The mass in this case - a huge left broad ligament fibroid with extreme vascularity, dense adhesions, prior three surgeries, and posterior bowel adhesions - created intraoperative conditions of significant difficulty. The ureteric transection occurred below a vascular clamp during haemostasis from the lateral wall.

This case is distinguished not by the occurrence of injury but by the response to it. The injury was identified immediately, and a successful ureteroureterostomy was performed by the combined team with DJ stent insertion. Both ureteric ends were viable, and the anastomosis was tension-free. No urinary leakage was detected intraoperatively, and the patient made a full recovery.

Delayed recognition, which occurs in up to 70% of ureteric injuries, is associated with urinoma, sepsis, fistula formation, and irreversible renal damage [4,10].

A proposed protocol for ureteric safeguarding

Based on the cumulative experience of this series, we propose the following structured six-step protocol for ureteric safeguarding in complex gynaecological surgery involving large fibroids.

Step One

Preoperative risk stratification: All patients with fibroids ≥ 8 cm, broad ligament or cervical fibroids, or prior pelvic surgeries should be identified as high risk.

Step Two

Imaging: Ultrasonography is the first-line modality. MRI pelvis with fibroid mapping should be performed to understand the relationship between the fibroid (e.g. broad ligament fibroid) and the ureter. Three-dimensional ureteric reconstruction by CECT should be performed in suspected large central cervical and lower segment fibroids.

Step Three

Prophylactic DJ stenting: Selective preoperative DJ ureteric stenting should be performed in cases with documented ureteric dilatation or displacement on imaging, or where difficult intraoperative ureteric identification is anticipated. A failed stenting attempt should be interpreted as diagnostic of significant ureteric compromise and should increase intraoperative vigilance accordingly.

Step Four

Intraoperative strategy: Enucleation of large cervical fibroid before uterine artery ligation should be considered​​​​​. In laparoscopic surgeries with an unusual location of fibroid obstructing the view of the uterine artery, intracapsular myomectomy is preferable before proceeding with hysterectomy. Systematic retroperitoneal entry through the space between the infundibulopelvic ligament and the round ligament, with identification and tracing of the ureter at the pelvic brim, should be performed in all high-risk cases. Direct ureteric visualisation should be maintained throughout critical dissection steps. Energy devices should not be used in proximity to identified ureteric structures.

Step Five

Adjunctive intraoperative verification: Intraoperative cystoscopy with confirmation of bilateral ureteric jets should be performed at the conclusion of the procedure in high-risk cases where DJ stents are not in situ. Indigo carmine or methylene blue dye may be used to facilitate this.

Step Six

Preparedness for injury: The surgical team should be mentally prepared for the possibility of ureteric injury in all high-risk cases. Urological assistance should be available on standby. Immediate intraoperative recognition and definitive repair are essential to optimising outcomes.

## Conclusions

Ureteric injury in complex gynaecological surgery may be substantially mitigated through a structured, anatomy-based approach integrating risk stratification, advanced imaging, selective stenting, and systematic retroperitoneal dissection and ureteric identification. Three-dimensional imaging provides valuable anatomical roadmapping in cases of distorted pelvic anatomy, while failed stenting signals ureteric compromise, warranting heightened intraoperative vigilance (Case 4). When injury occurs despite precautions, immediate intraoperative recognition and definitive repair remain the most critical determinants of outcome.
